# A Ranging System Integrating Time‐of‐Flight Approach With Optical‐Frequency‐Comb Interferometry for Ultra‐Precise Long‐Distance Measurements

**DOI:** 10.1002/nap2.70343

**Published:** 2026-11-11

**Authors:** Cunliang Yang, Xiao Wang, Zihao Zhang, Junhao Li, Jingjing Wang, Jinman Ge, Lizhong Xiao, Jijun Feng

**Affiliations:** ^1^ Shanghai Key Laboratory of Modern Optical System, Engineering Research Center of Optical Instrument and System (Ministry of Education) School of Optical‐Electrical and Computer Engineering University of Shanghai for Science and Technology Shanghai China; ^2^ Institute of Advanced Precision Spectroscopy of East China Normal University Shanghai China; ^3^ National Key Laboratory of Electromagnetic Space Security Tianjin China; ^4^ National Key Laboratory of Science and Technology on Space Microwave China Academy of Space Technology Xi’An China; ^5^ School of Computer Science and Information Engineering Shanghai Institute of Technology Shanghai China

**Keywords:** flight time method, high precision absolute ranging, optical frequency comb, pulse interference autocorrelation

## Abstract

Time‐of‐flight (TOF) ranging and pulse interference have been widely applied in high‐precision absolute ranging due to high robustness and low ambient noises. However, TOF is typically limited by low resolutions, whereas the absolute lengths determined using the pulse interference method are discrete. In this study, an absolute ranging system integrating TOF with OFC‐based interferometric modules was developed. In this system, the TOF module provides a rapid yet rough distance measurement, whereas the Michelson interferometry module significantly improves the ranging accuracy using interference techniques. First, the approximate distance was estimated using the TOF laser rangefinder. Then, the spatial position information of femtosecond pulse interference signals was determined through OFC‐based interferometric measurement. Finally, the exact distance was determined based on the rough measurement and the fine measurement. Sub‐meter simulations and practical measurements revealed that the t‐distribution‐based limit error were ± 1.3 and ± 2 μm within 50 m, respectively, demonstrating that the proposed ranging system integrating pulse coarse with OFC achieved a micron‐level absolute distance accuracy for 10‐m‐scale measurement tasks. The proposed system can be further optimized in terms of active frequency stabilization, extended TOF range, and full‐wave sampling and processing. This study paves the way for effective long‐distance ranging with ultra‐high precision requirements.

## Graphical abstract

A TOF and OFC interferometry integrated ranging system uses TOF for rough distance and Michelson interferometry for fine femtosecond‐pulse measurement. Sub‐meter simulations and practical measurements yielded t‐based limit errors ± 1.3 μm and ± 2 μm within 50 m, enabling micron‐level absolute ranging at 10‐m scale. This enables ultra‐high precision long‐distance ranging.

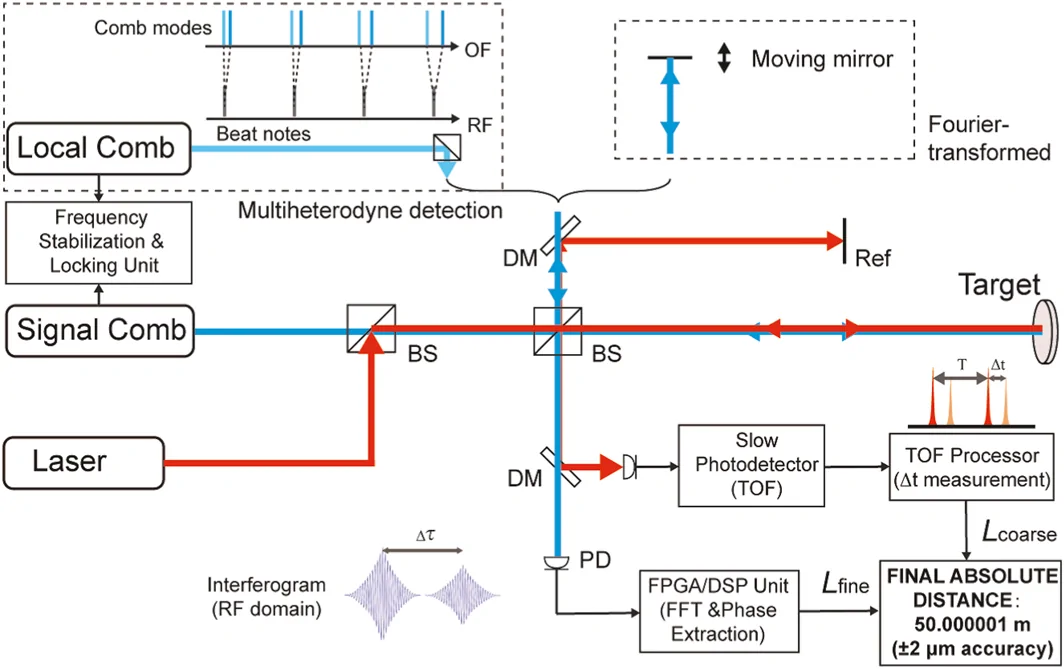

## 1 Introduction

High‐precision absolute ranging plays a critical role in satellite monitoring, large‐surface shape profiling, and advanced industrial manufacturing. It can be achieved using active optical measurement techniques by reconstructing the object surface through the projection of predefined light points or images based on reflected light [1, 2, 3]. Among these methods, the geometric approaches capture illumination patterns on the object surface using a camera [4]. By accurately modeling the camera and illumination coordinate systems, the spatial coordinates of each point on the illuminated object can be reconstructed via triangulation. Hence, the geometric approaches are essentially a cost‐effective solution for rapid ranging where the precision requirement is not extreme [5, 6]. The time‐of‐flight (TOF) method determines distances by measuring the delay between the emission of a light pulse and its reception by a detector, and this process requires a high bandwidth to achieve precise timing [7, 8]. Nevertheless, intensity‐modulated techniques rely on phase modulation of light signals to reduce range blur, and flight time is determined by calculating the corresponding phase shifts [9].

For measurements requiring micro‐ and nanoscale precision, interferometric methods (e.g., single‐wavelength phase‐shifting interferometry, coherent scanning interferometry, and swept‐frequency interferometry) are typically employed [10]. However, these approaches have intrinsic drawbacks. For instance, the effectiveness of single‐wavelength interferometry for complex surface detection is limited by phase ambiguity [11]. Coherent scanning interferometry exhibits a limited measurement field and low spatial coherence, despite that its light sources have low coherence [12]. Swept‐frequency interferometry tends to have a limited measurement range due to the depth‐of‐focus constraints, although it is featured by fast scanning and wide field of view [13]. It has been demonstrated that measurement accuracy of interferometric methods is highly dependent on the characteristics of the light source [14, 15]. For instance, the uncertainty of TOF measurements is closely related to the timing jitter, the phase resolution of TOF measurements is affected by the laser linewidth, and the field of view of TOF measurements depends on the spatial coherence [16, 17, 18]. As the measurements become increasingly complex, traditional methods fail to meet the high‐precision requirements of modern engineering applications.

In TOF measurements, absolute distances were determined based on the time delay between emitted and received pulses during propagation. However, the accuracy of TOF is at the millimeter scale due to restrictions by the response time of the detector [19]. Pulse‐sequence interference can improve measurement accuracy to the nanometer scale, whereas its measurable distance is confined to half of the wavelength range. As a result, the results delivered by pulse‐sequence interference tend to be discrete [20, 21]. Proposed in the early 2000s, optical frequency comb (OFC) enables direct conversion between optical and radio frequencies, thus facilitating frequency tracking, enhancing frequency stability, and providing an ultra‐wide spectral range [22]. Therefore, OFC has been widely applied in optical atomic clocks, quantum information technologies, advanced manufacturing, defense, and space‐based laser communications [23, 24, 25], providing effective solutions for high‐precision ranging and distance measurement.

In this study, a TOF‐assisted OFC ranging system was developed for large‐range high‐precision absolute distance measurement. In this system, the TOF module provides rapid and timely rough distance measurements, whereas the OFC‐based interferometric modules significantly improve the ranging accuracy. Specifically, the approximate distance was estimated using the TOF laser rangefinder. Then, the spatial position information of femtosecond pulse interference signals was determined through OFC‐based interferometric. Additionally, the exact distance was determined based on estimation by the TOF method and measurement by the interferometry module. Sub‐meter simulations and practical measurements were performed, and the results were compared to each other to validate the proposed system. The main contribution lies in integrating large‐range TOF ranging with complementary OFC‐based precision measurements within a unified ranging framework.

## 2 Theory

The basic distance in a TOF system can be calculated by the following equation [26]:

(1) $$d=\frac{c\Delta t}{2n_{g}},$$

where *n*_*g*_ is the group refractive index of air and Δt is the round‐trip time and *c* is the speed of light.

Although suitable for long‐range measurements, this equation shows inherently limited accuracy due to the timing resolution of the detector and the width of the light pulse. To improve the ranging precision, a single‐comb Michelson interferometer is introduced. The optical frequency of the *qth* comb line is $\nu_{q}=f_{\text{ceo}}+qf_{r}$, and the detected interference signal is as follows [27]:

(2) $$I\left(\Delta L\right)=I_{\text{dc}}+2\sum_{q}\sqrt{P_{r,q}P_{m,q}}\;\cos\left[\frac{4\pi }{c}\left(f_{\text{ceo}}+qf_{r}\right)\Delta L+\phi_{q}\right],$$

where, *I*_*dc*_ is the background, *P*_*r,q*_ and *P*_*m,q*_ are the powers of the *qth* comb line in the reference and measurement arms, Δ*L* is the one‐way optical‐path mismatch within the selected pulse‐overlap order, and *φ*_*q*_ is the residual phase. The coherent comb‐line sum forms carrier fringes within a pulse‐interference envelope; fitting its maximum yields the fine distance.

Dual‐comb ranging can also overcome these limitations by using two OFC with slightly different repetition frequencies $\Delta f_{r}$. After a probe pulse is reflected from the target, two separate pulse trains with a time delay of Δτ are sampled linearly by the pulse train from reference comb. Owing to the small repetition‐rate difference, the optical delay is temporally magnified in laboratory time as follows:

(3) $$\Delta t=b\cdot \Delta \tau ,b=\frac{f_{r}}{\Delta f_{r}},$$

where, *b* is the temporal magnification factor, Δt is determined by the peaks of the fitted envelopes [28]. Therefore, the distance can be obtained from Equation (1), expressed as follows:

(4) $$d_{\text{dual}-\text{comb}}=\frac{c}{2n}\Delta t=\frac{c}{2n}\times \frac{\Delta f_{r}}{f_{r}},$$

By precisely retrieving the interferometric phase [29, 30], Δt can be further refined, enabling dual‐comb ranging to combine long‐range measurement with higher precision.

The absolute geometric distance is then expressed as follows:

(5) $$D=x_{\text{pk}}+\frac{mc}{2n_{g}f_{r}},$$

where *D* is the absolute distance, $x_{\text{pk}}$ is the fine residual distance obtained from the fitted interference‐envelope maximum, *m* is the integer pulse order, *f*_*r*_ is the comb repetition frequency, and *x*_pk_ is obtained from the fitted envelope maximum.

Because the cosine term in Equation (2) is periodic, the interferogram alone cannot determine *m*. The coarse TOF result from Equation (1) is therefore used to select the nearest pulse order:

(6) $$m=\text{round}\left(\frac{d}{L_{\text{rep}}}\right),L_{\text{rep}}=\frac{c}{2f_{r}},$$

where round(·) denotes rounding to the nearest integer and $L_{\text{rep}}$ is the comb‐defined one‐way optical‐path interval.

The air correction is evaluated from the group refractive index [31, 32, 33]:

(7) $$\begin{array}{c} n_{p}\left(\lambda ,T,p,H,x_{c}\right)-1=\frac{\rho_{a}}{\rho_{a,s}}\left[n_{a,s}\left(\lambda ,x_{c}\right)-1\right]+\frac{\rho_{w}}{\rho_{w,s}}\left[n_{w,s}\left(\lambda \right)-1\right],\\ n_{g}\left(\lambda ,T,p,H,x_{c}\right)=n_{p}\left(\lambda ,T,p,H,x_{c}\right)-\lambda \frac{\partial n_{p}}{\partial \lambda } \end{array}$$

where, *n*_*p*_ and *n*_*g*_ are the phase and group refractive indices; *T*, *p*, *H,* and *x*_*c*_ are temperature, pressure, relative humidity, and CO_2_ concentration; *ρ*_*a*_ and *ρ*_*w*_ are the dry‐air and water‐vapor densities; and the subscript *s* denotes reference conditions.

## 3 Simulation and Modeling

The key components in this system are the TOF module and the Michelson interferometer module, which enable self‐correlation of the flight‐time pulse interference.

### 3.1 Distance Measurement Time Module

Figure 1 illustrates the optical path of the proposed ranging system. As indicated, the system is centered on a standard Michelson interferometer optical path, with an OFC laser source emitting femtosecond pulse. The pulse laser beam passes through a collimating mirror and an expanding mirror before being split by a beam splitter. One beam is reflected and returns to the beam splitter via a reference plane mirror; this beam serves as a reference beam. The transmitted beam is reflected from a reflector positioned at the target object, and this beam serves as a measurement beam. Eventually, the reference and measurement beams are integrated and detected by the avalanche photodiode (APD). The TOF module provides a rough distance measurement at the target position, and the result is then integrated with the Michelson interferometer analysis to deliver the precise absolute distance.

**FIGURE 1:**
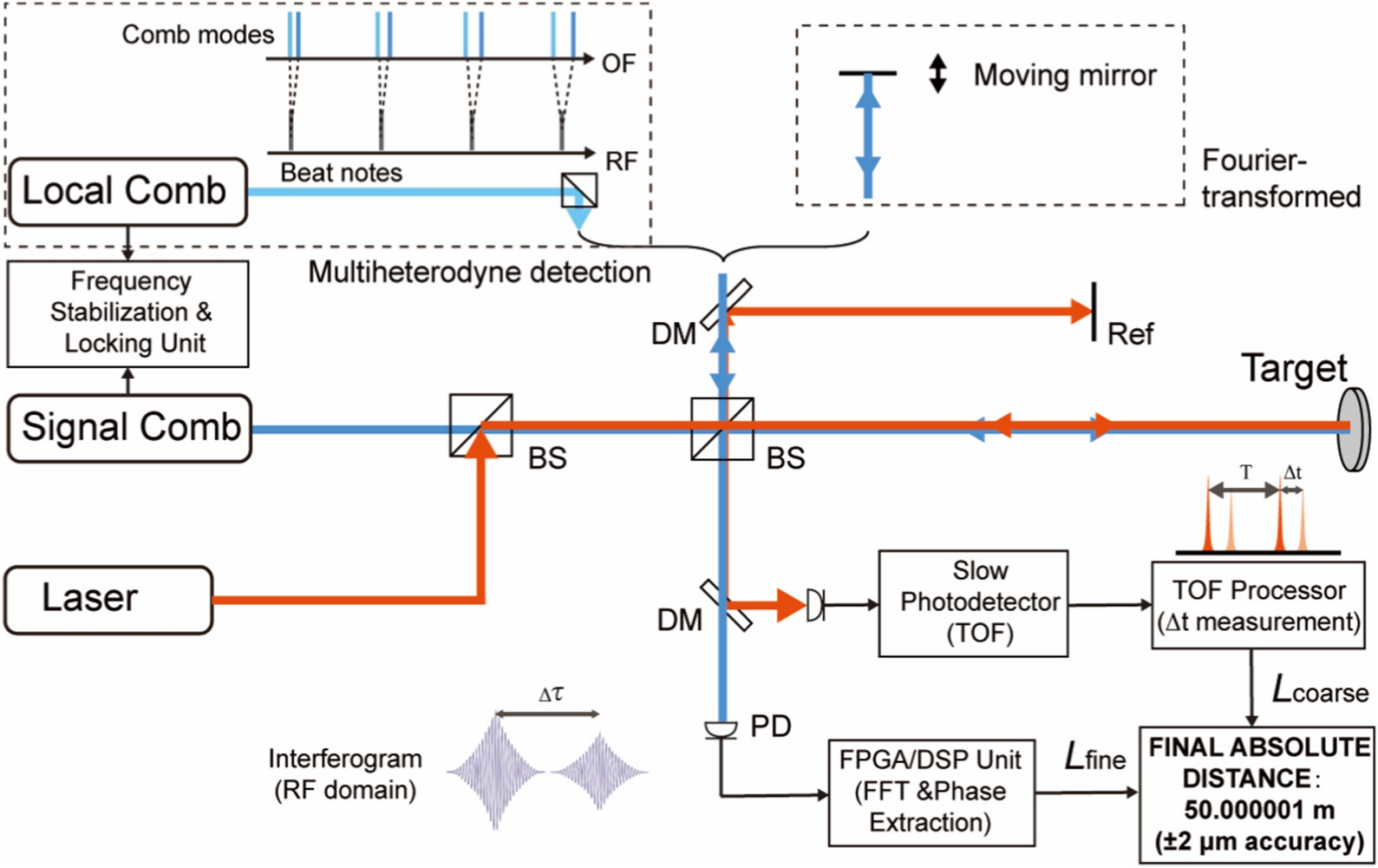
Schematic of the optical path for ranging by the proposed ranging system. BS, beam splitter; DM, dichroic mirror; PD, photodetector.

As mentioned, the TOF ranging method determines the target distance based on the flight time of a pulse between the signal and the reflection surface of the light source. In non‐line‐of‐sight environments, distances estimated based on signal strength indicators are reliable and preferred. In line‐of‐sight environments, the TOF ranging method can resolve distance measurements that cannot be accurately determined using signal intensities alone [34]. In the TOF ranging method, it is essential to ensure both synchronization and consistency of the transmitter and receiver, as well as accurate control over the signal transmission timing of the receiver unit.

As shown in Figure 1, the laser rangefinder in the flight time module serves as the detector. The continuous laser that produces visible light (red) at a wavelength of 650 nm was employed as the laser emitter. Consequently, the measurement range was 0.1–100 m, and the sampling frequency was 50 Hz. Also, the measurement accuracy was ± 2 mm, and the minimum resolution was 0.1 mm. Overall, the laser rangefinder can provide a coarse measurement of the distance, thereby allowing determination of the integer multiple *m* of the pulse interval, eventually facilitating rapid measurement in subsequent procedures.

### 3.2 Michelson Interferometer Module

Interference measurement has been widely used for high‐precision ranging due to its non‐contact nature, high accuracy, and good sensitivity [35, 36]. Common interferometers include Michelson, Mach–Zehnder, and Fabry–Perot, and Michelson interferometer is typically employed for the measurement of length and refractive index. In this method, laser‐coherent ranging is conducted using a Michelson interferometer. Specifically, interference is achieved by tuning the separation and reunion of the coherent light. Continuous adjustment of angle and/or position of the reference mirror generates equal‐thickness or equal‐inclination interference fringes. In a Michelson interferometer, a laser emits coherent light into a beam splitter, which reflects and transmits the light to generate reference and measurement beams that are perpendicular to each other. These beams are reflected by mirrors along their respective optical paths and return to the beam splitter for coherent interference, resulting in detectable light and dark interference fringes. Interference fringes are the trajectories of points on the receiver surface with an identical optical path difference. The optical path difference can be adjusted by tuning the length of the interference arm or the refractive index of the medium. As a result, a variety of interference patterns with different shapes can be generated on the CCD detector.

A Gaussian pulse with a central wavelength of 800 nm and a pulse width of 94 nm was used as the light source. The reference mirror was fixed at 20 mm in the reference optical path, whereas the measurement mirror was fixed at 20 mm in the measurement optical path and independently displaced at a step of 1 μm. In this case, Figure 2 shows the equal‐inclination interference fringes used to calibrate the absolute zero position of the movable reference mirror.

**FIGURE 2:**
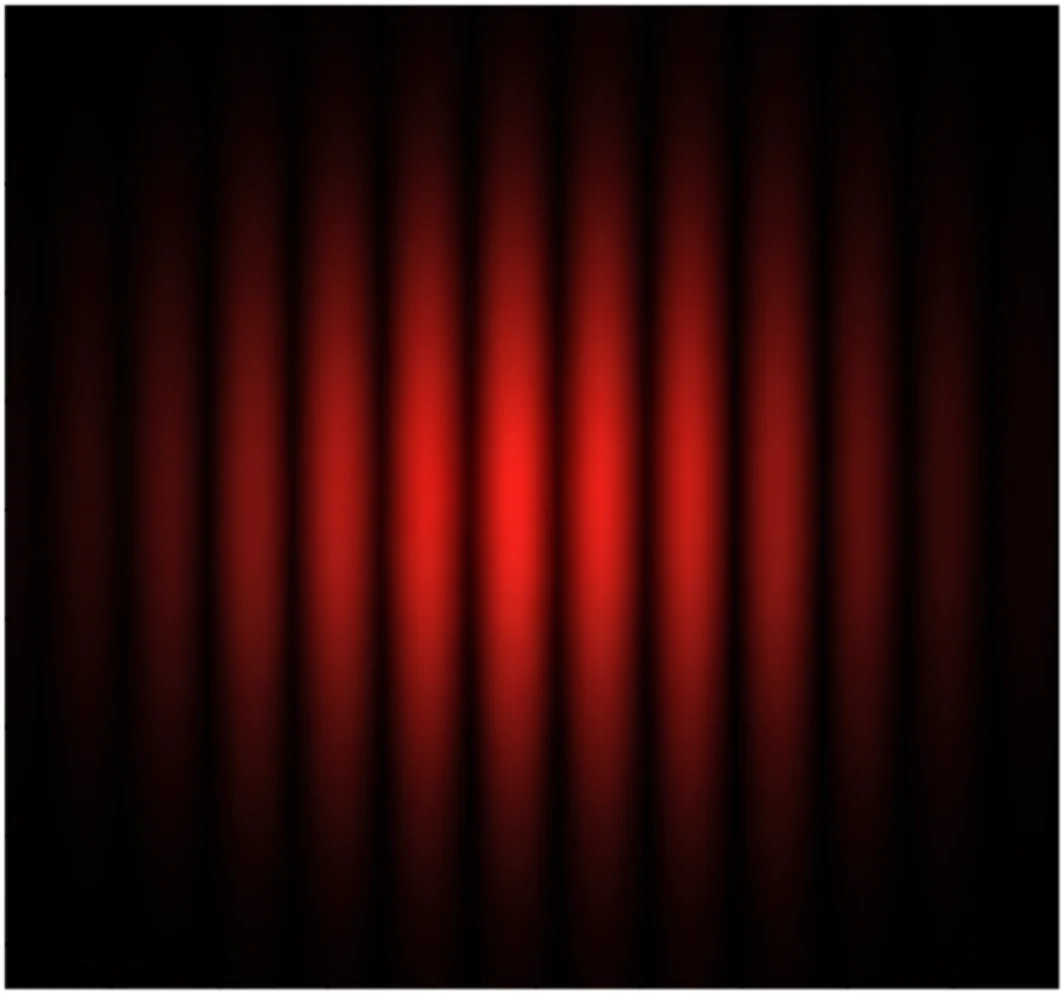
Interference fringes induced by the optical path difference between the reference and measurement paths.

The interference pattern of the integrated light with a clear brightness distribution and uniformly distributed fringes is captured by the camera. Then, intensity distribution of the interference pattern was measured using a radiation illumination detection unit. Figure 3a shows the radiation intensity distribution of the interference pattern before interpolation fitting, whereas Figure 3b shows the radiation intensity distribution of the interference pattern after interpolation fitting. As indicated, the peak was located at 39.0625 μm forward from 0. The Michelson interferometer component of the absolute ranging task was simulated and analyzed.

**FIGURE 3:**
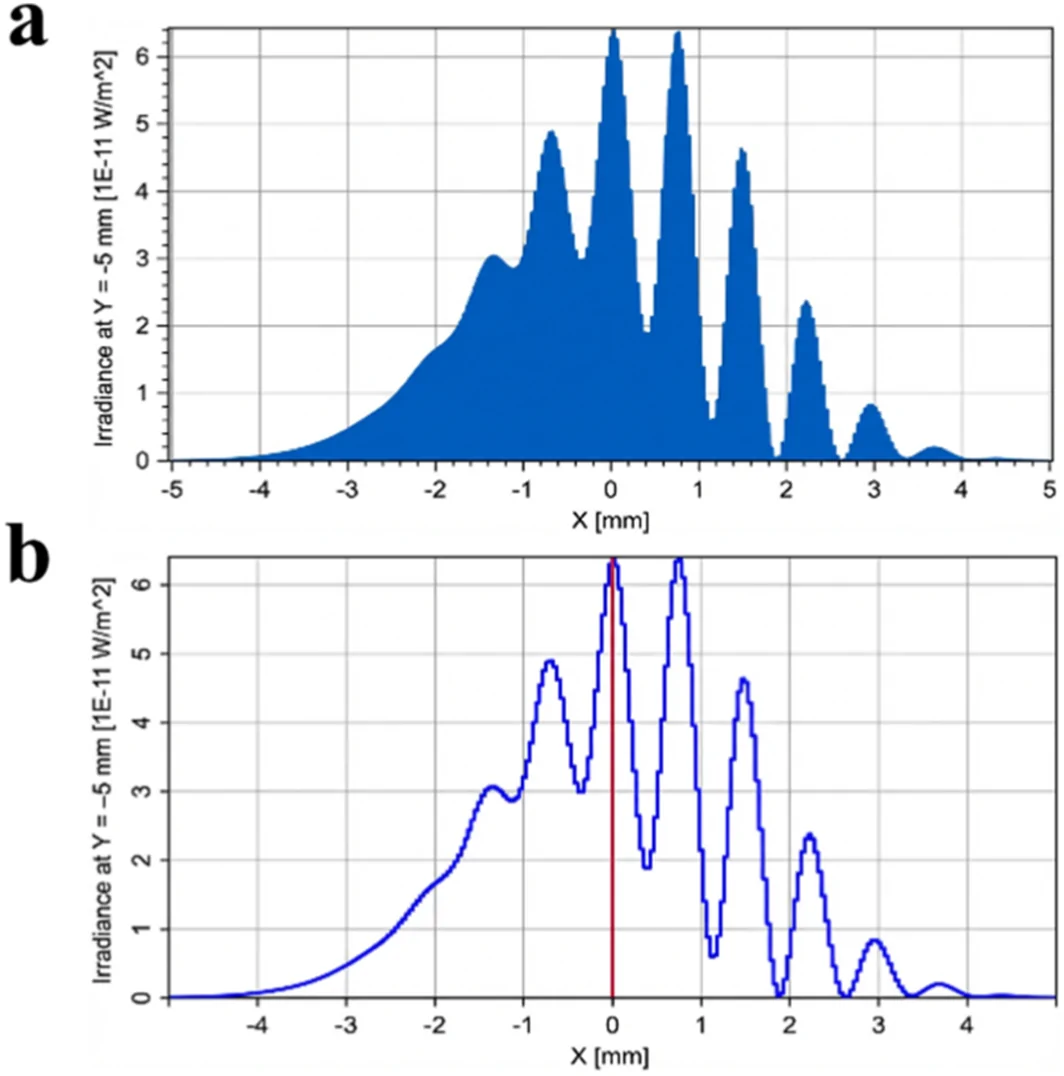
Radiation intensity distribution of the interference pattern (a) before and (b) after interpolation fitting.

Figure 4 shows the optical path in the Michelson interferometer. As indicated, the optical path difference between the reference and measurement paths varied as the position of the reference mirror changes. Consequently, changes were detected in the interference pattern received by the detector and the radiation intensity of the interference signal, thus allowing monitoring of the pulse interference autocorrelation fringes at each scanning site. As scanning continued, the optical path difference varied with the length of the reference optical path, resulting in variation in the intensity information of the electric field component carried by the interference fringes. In this study, only interference autocorrelation fringes at different sampling sites on the reference mirror were observed (Figure 5).

**FIGURE 4:**
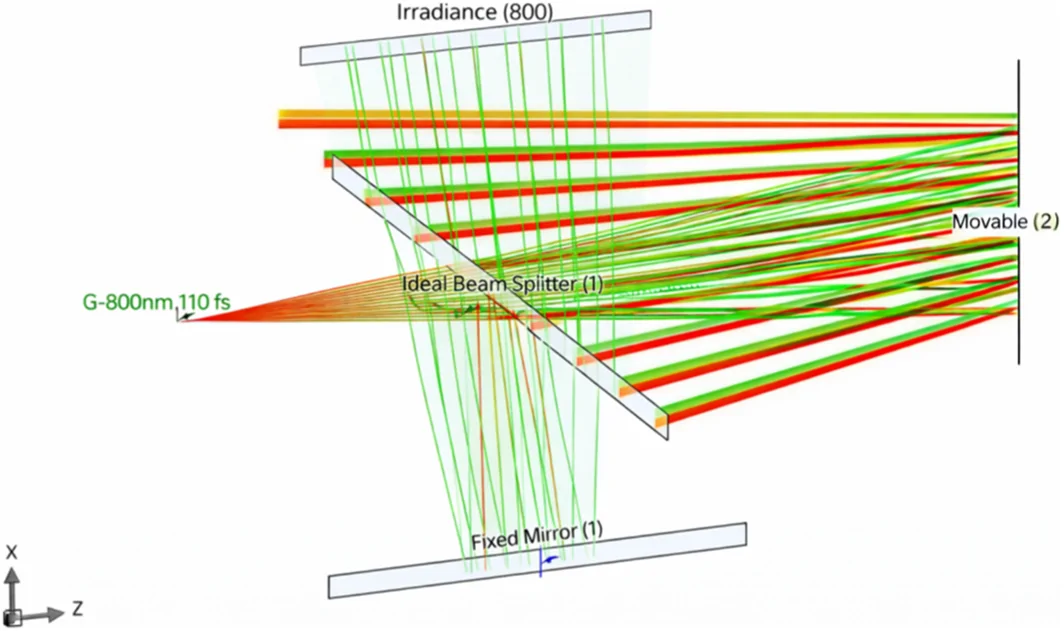
Optical path in the Michelson interferometer.

**FIGURE 5:**
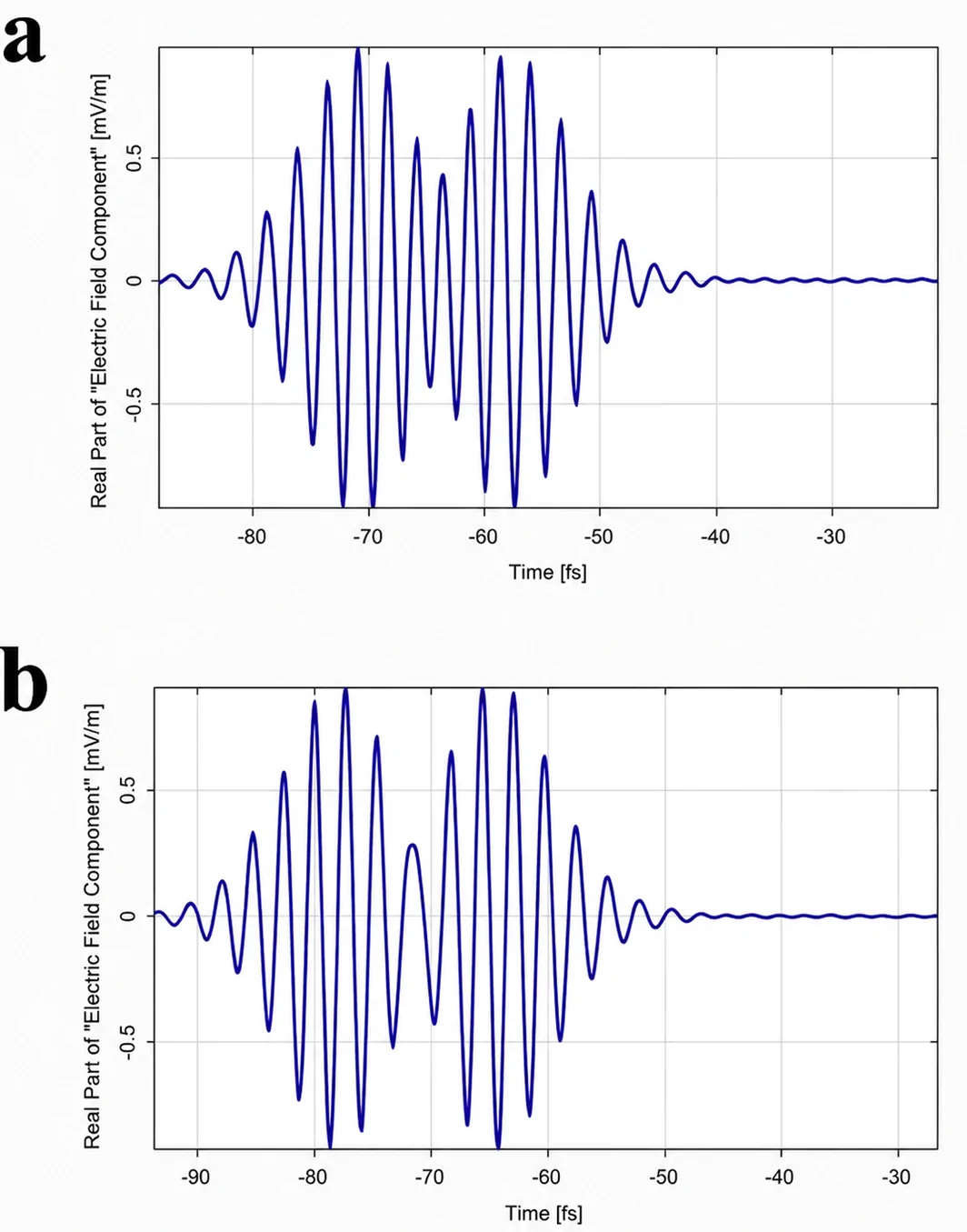
Interference autocorrelation fringes at (a) 19.999 mm and (b) 20.001 mm.

Figure 5a,b show the interference autocorrelation fringes at 19.999 and 20.001 mm on the reference mirror, respectively. The distance to be measured was calculated by identifying the position of the peak intensity of the interference signal, which corresponds to the optical path difference between the reference and the measurement optical paths. By tuning the repetition frequency and the optical comb, multiple measurements were performed at the same site to minimize random errors. Femtosecond pulse interference autocorrelation waveform measured in the Michelson interferometer module (Figure 6). The recorded interferometric signal (carrier fringes) is post‐processed by a low‐pass sliding RMS operation to obtain a smooth envelope, and the envelope peak is identified (maximum sample) as the delay corresponding to the pulse‐overlap condition. The peak delay is mapped to the optical path difference (OPD) between the reference and measurement arms, enabling absolute distance retrieval when combined with the coarse TOF prior and repeated measurements for uncertainty reduction.

**FIGURE 6:**
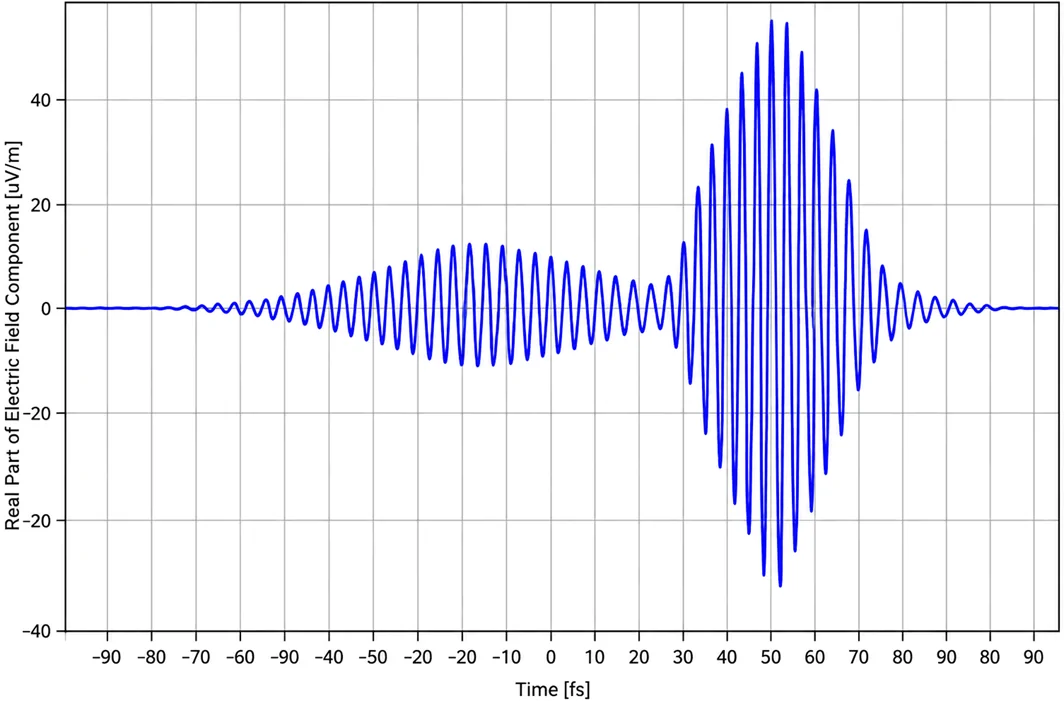
Results of femtosecond pulse interference autocorrelation.

## 4 Results and Discussion

For the Michelson autocorrelation interference measurement, the pressure and temperature were maintained at 101.325 kPa and 20°C, respectively. The repetition frequency of the optical frequency comb source was fixed at 100 MHz, the scanning step was 0.1 Hz, and a total of 11 sampling sites were involved. Figure 7 shows the pulse interference autocorrelation result obtained using the detector at a repetition frequency of 100 MHz. The results confirmed basic characteristics of femtosecond laser pulse autocorrelation delay. Also, the relative distance (D) of interference fringes can be determined based on from the time delay between the reference and measurement pulses.

**FIGURE 7:**
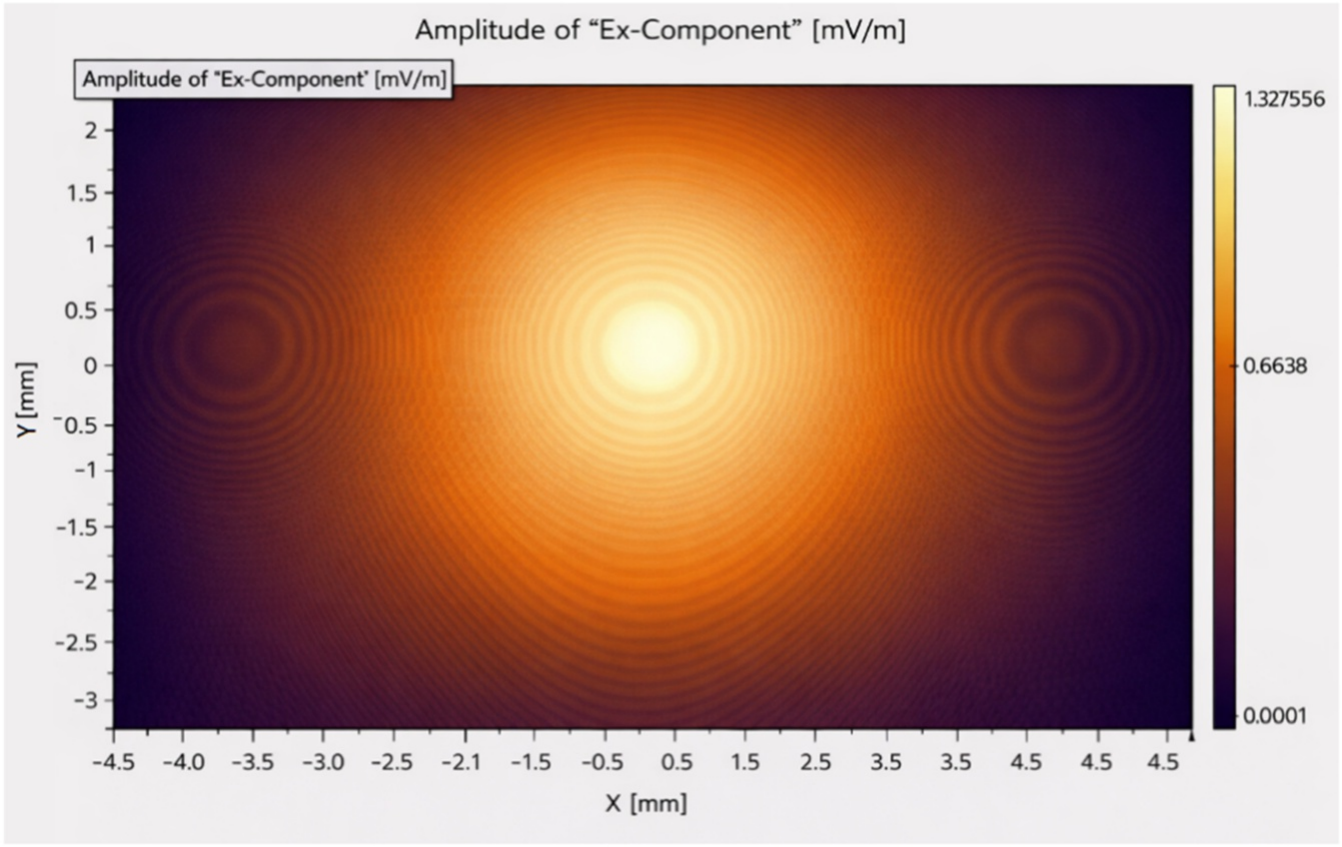
Detection results obtained using the photoelectric detector.

Table 1 lists the detection results at different sampling sites obtained using the photoelectric detector. The results included the first peak moment (Moment 1), the adjacent second peak moment (Moment 2), the time interval between them, and the corresponding distance *L*_i_. Correct order assignment requires the TOF error to remain below $L_{\text{rep}}/2$. At $f_{r}=100$ MHz, the decision boundary is approximately 0.749 m, far larger than the ±2 mm TOF error. An error of one pulse order would introduce a distance offset of $c/\left(2n_{g}f_{r}\right)\approx 1.499$ m. The TOF result of approximately 2 m therefore gives *m* = 1 for all 11 measurements in Table 1. The listed *L*_i_ values are the final absolute distances obtained by combining this pulse order with the residual distance measured by pulse‐interference autocorrelation. For the estimation of the 95% confidence interval (95% CI), normal distribution and t‐distribution are typically employed in cases of large and small sample sizes, respectively. In this study, the t‐distribution was used to determine the limit error of the arithmetic mean. With a degree of freedom (DOF) of *υ* = *n*‐1 = 10 and a significance level of *α* = 0.05, t*α* was determined to be 2.23. The limit error δlim *x* of the arithmetic mean was calculated by the following equation:

(8) $$\delta \;\lim\;x=\pm t_{\alpha }\times \sigma_{x}=\pm 2.23\times 0.0000006m\approx \pm 0.0000013$$

**TABLE 1:** Detection results at different sampling sites obtained using the photoelectric detector.

Sample site	Moment 1 (ns)	Moment 2 (ns)	Time interval (ns)	*L*_i_ (m)
1	14.314415	20.981092	6.666677	2.0000031
2	14.317092	20.983754	6.666662	1.9999986
3	14.320132	20.986794	6.666662	1.9999986
4	14.323361	20.990033	6.666672	2.0000016
5	14.326924	20.993588	6.666664	1.9999992
6	14.327111	20.993774	6.666663	1.9999989
7	14.330165	20.996837	6.666672	2.0000016
8	14.333782	21.000447	6.666665	1.9999995
9	14.336541	21.003213	6.666672	2.0000016
10	14.339884	21.006562	6.666678	2.0000034
11	14.342113	21.008792	6.666679	2.0000037

The measured absolute distance can be expressed as follows:

(9) L=x+δlimx=(2.0000009±0.0000013)m

The feasibility of the proposed ranging system was verified by simulations. The concept of residual error, defined as the difference between the measured distance and the actual distance, was employed in this case. According to the simulation results, the cm‐scale absolute distance was determined to be *L* = (2.0000009 ± 0.0000013) m, with a limit error of ± 1.3 μm. The peak‐to‐peak limit error (*r*) did not exceed 2.6 μm, and no gross errors were detected, demonstrating good feasibility of proposed ranging system integrating incoherent and coherent ranging approaches in high‐accuracy absolute distance measurements.

## 5 Recommendations for Improvements

Despite great advantages reported above, dual‐comb ranging was conducted to further improve the proposed ranging system. The improvement was realized in three aspects, including active frequency stabilization, extended TOF range, and full‐wave sampling and processing.

### 5.1 Active Frequency Stabilization

Dual‐comb ranging can realize micrometer‐ or even nanometer‐level accuracy, provided the repetition frequency remains stable [37, 38]. The active frequency stabilization unit comprises two optical combs (reference and sample), I/V conversion circuits for both combs, signal filters and amplifiers, mixers, and PZT (piezoelectric ceramics) drivers, as shown in Figure 8. Herein, the signal generator produces two mixer reference signals: one goes to the reference mixer at the 5th harmonic of reference comb repetition, whereas the other is directed to the 2nd stage mixer with a frequency equal to the difference between the inter‐comb repetition. To correct the error in the repetition rate of reference and sample combs, two error signals are output by each mixer. Then, these signals drive two PZTs, which adjust the resonant length of the optical frequency comb. Figure 9 shows the errors with and without frequency stabilization. As indicated, the error was drastically reduced with frequency stabilization, suggesting that active frequency stabilization can effectively minimize errors and improve measurement accuracy.

**FIGURE 8:**
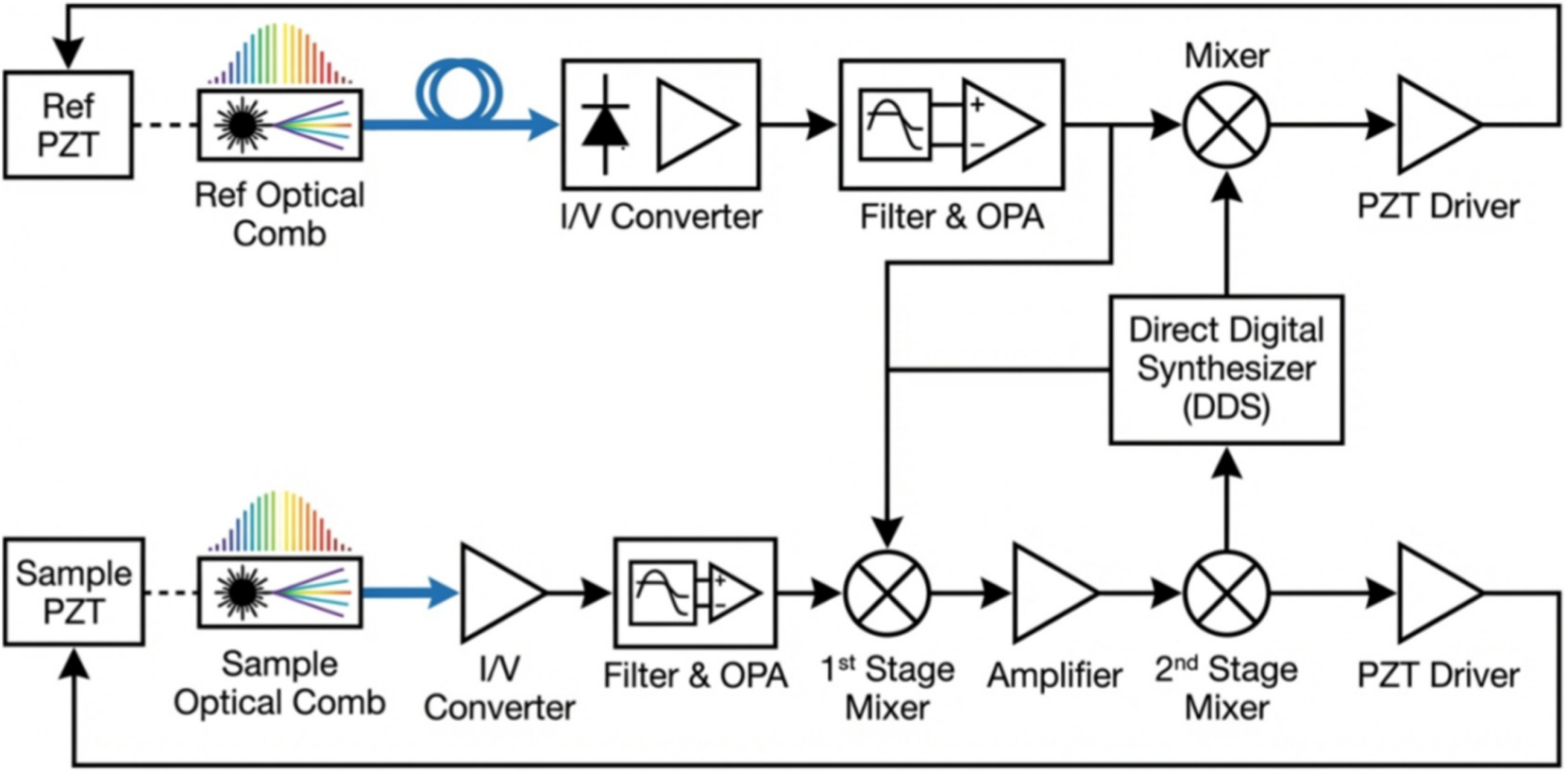
Schematic of the active frequency stabilization unit.

**FIGURE 9:**
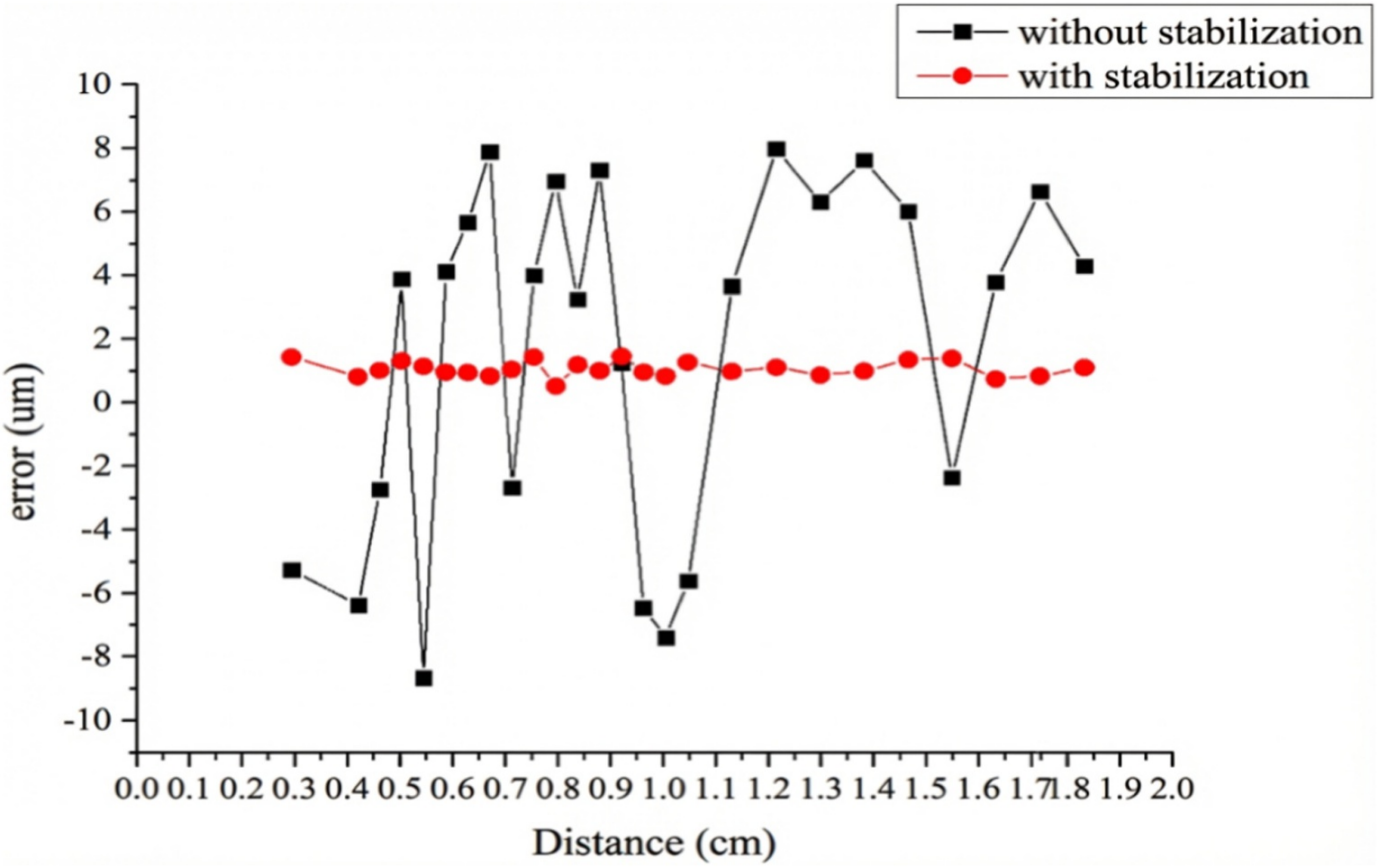
Measurement errors with and without frequency stabilization in the range of 0.3–1.85 cm.

### 5.2 Extended TOF Range

To achieve 50‐m ranging, TOF was employed for rough measurement. As shown in Figure 10, DFB laser with a wavelength of 1064 nm and a power of 10 dbm was used in the transmit chain. The TOF receiver converted the 1064‐nm echo pulses into signals suitable for post‐processing. The rough ranging receiver consisted of a photodetector, preamplifier, AGC, constant‐ratio timing, and comparators. To ensure accurate rough ranging, the constant‐ratio timing unit function was used as the photodetector which required a high‐voltage bias supply. The receiver had a bandwidth of 120 MHz and a dynamic range exceeding 40 db.

**FIGURE 10:**
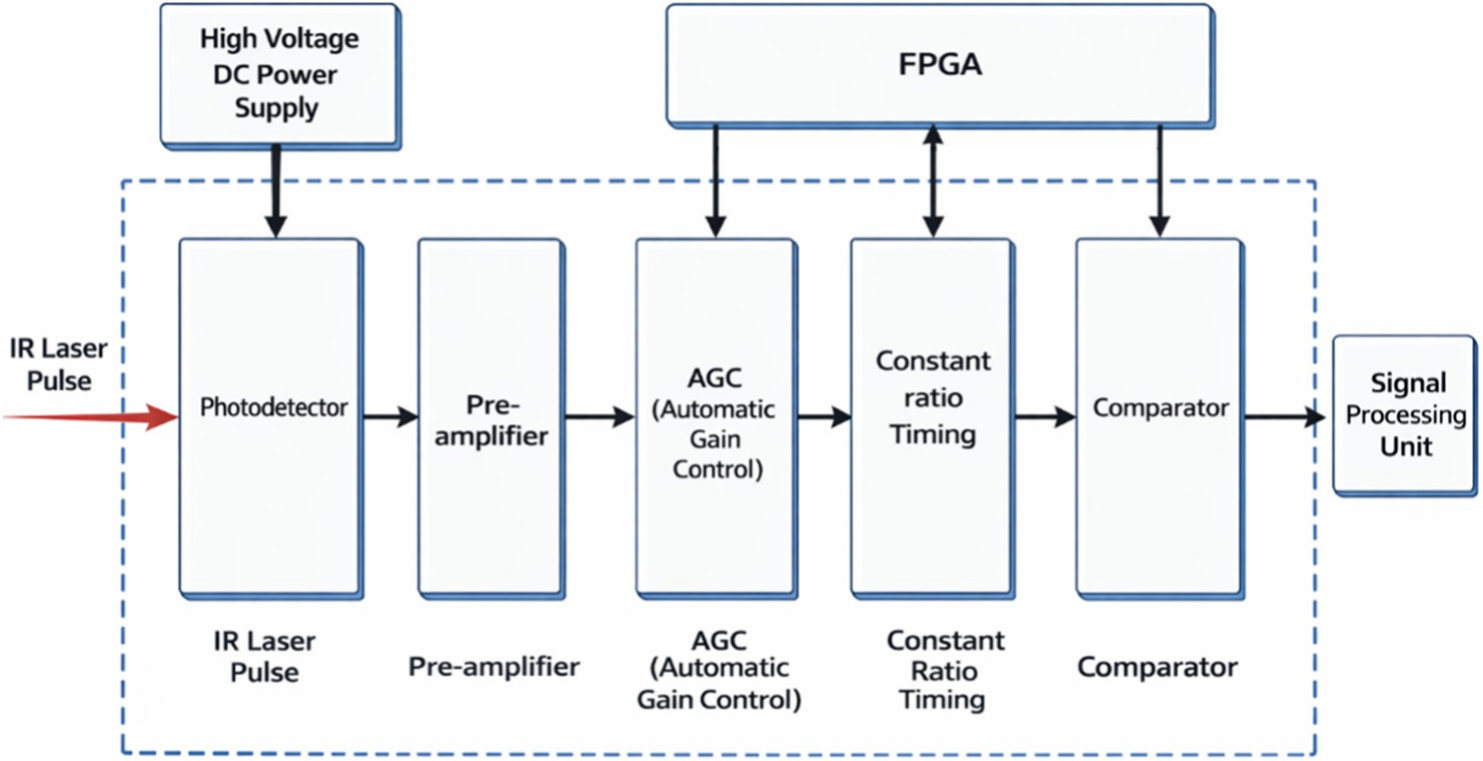
Procedures of rough ranging.

### 5.3 Full‐Wave Sampling

Full‐wave sampling can determine distance based on target phase changes [39]. In this study, phase measurement was achieved using several methods, including delay calculation with a fast clock after comparator shaping and Fourier transform after ADC sampling. Although ADC sampling retained more echo information, it relies heavily on discrete Fourier transform (DFT) processing. To improve the updating speed, the radix‐4 fast Fourier transform was implemented on a customized circuit platform. Consequently, a 4096‐point Fourier transform can be completed within 100 ms. Figure 11 shows the algorithm used for full‐wave sampling and radix‐4 Fourier transform ranging. To validate the long‐distance ranging performance, we performed a 50 m experiment using a multi‐pass cavity. The cavity provided an effective 50 m optical path. We selected measurement points at different distances and performed repeated measurements to evaluate repeatability. Before the experiment, the zero‐position offset, reference‐arm position, comb repetition frequency, and air‐refractive‐index correction were calibrated. At each point, the TOF result determined the integer pulse order *m*, whereas dual‐comb ranging provided the residual distance. The experimental error was evaluated against the calibrated cavity distance. Figure 12 presents the measurement results at multiple distances over the full range up to 50 m. At each distance, repeated measurements were performed, and the t‐distribution was used to calculate the limit error of the arithmetic mean. This analysis provides a statistical evaluation of the measurement performance at different distances. The error remains within a few micrometers across the measurement range, and practical experiments show a maximum absolute error of approximately ± 2 μm within 50 m, consistent with the system‐level accuracy claim. The slowly varying error trend suggests that the residual is dominated by systematic/slow‐drift factors (e.g., repetition‐rate stability and environmental perturbations) rather than purely random noise, which agrees with the improvement observed when active frequency stabilization is applied. At 800 nm, 20°C, 101.325 kPa, 40% relative humidity, and 450 ppm CO_2_ give *n*_*g*_ ≈ 1.00027. Assuming path‐averaged fluctuations of 0.02°C, 10 Pa, 1% relative humidity, and 50 ppm CO_2_, the corresponding contribution is approximately 0.5–1 μm over 50 m and is included in the uncertainty budget. The modified Edlén equation is used only for comparison within its validated wavelength range.

**FIGURE 11:**
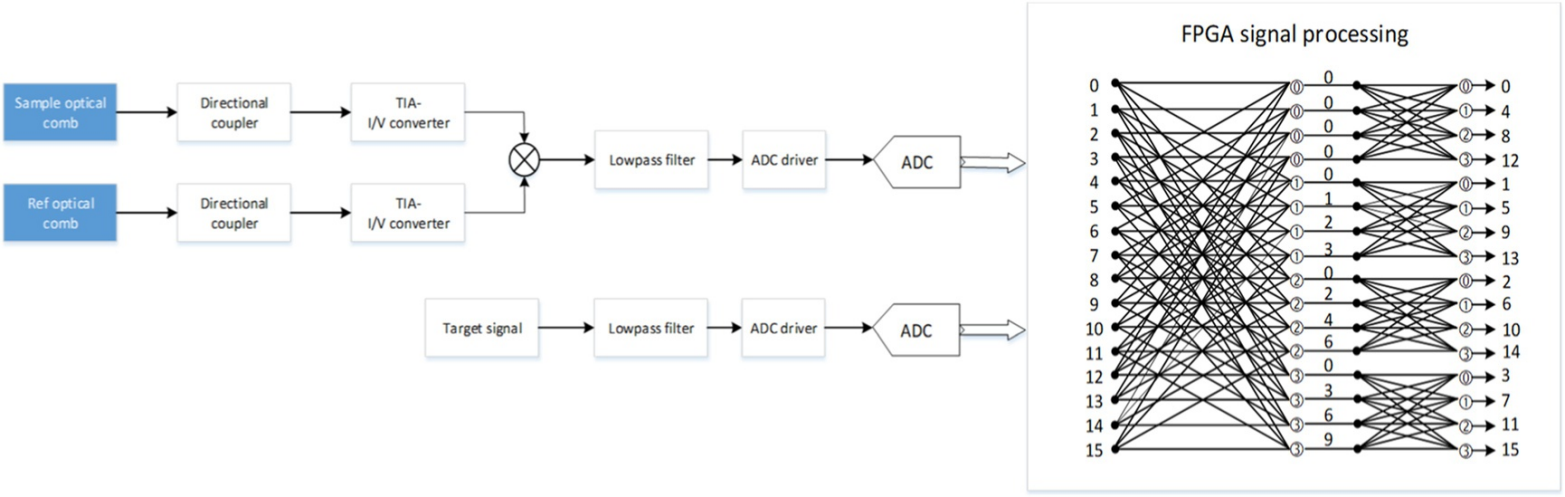
Algorithms for full‐wave sampling and ranging.

**FIGURE 12:**
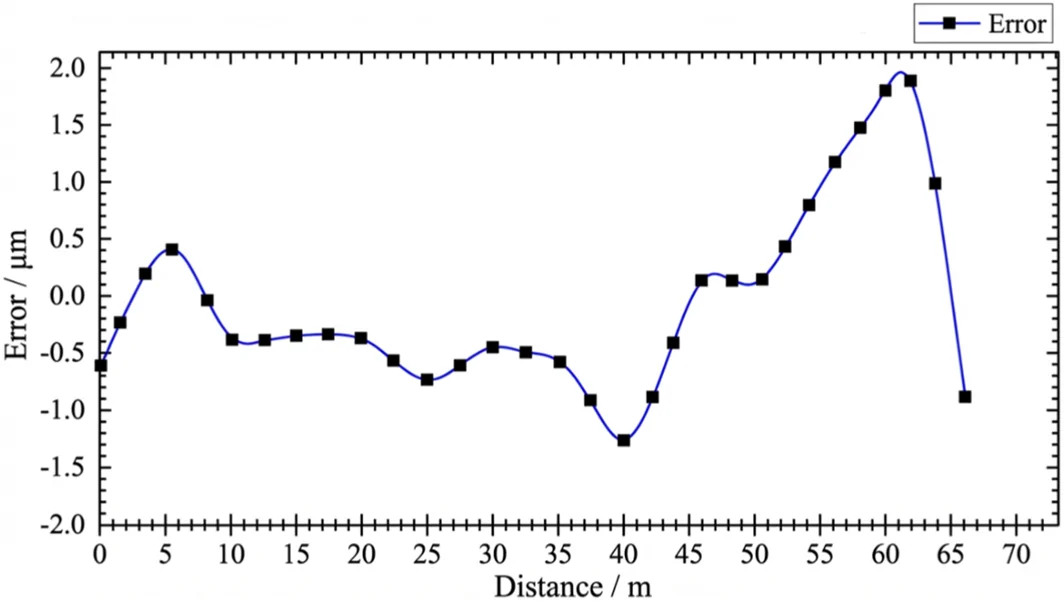
Ranging errors.

## 6 Conclusions

An absolute ranging system integrating TOF with OFC‐based interferometric measurement was developed. In this system, the TOF module provides a rapid yet rough distance measurement, whereas the Michelson interferometry module significantly improves the ranging accuracy using interference techniques. First, the approximate distance was estimated using the TOF laser rangefinder. Then, the spatial position of fs pulse interference signals was determined by interference measurement. Finally, the accurate distance was determined based on the results of both rough measurement and the fine measurement. Sub‐meter simulations and practical measurements revealed that the t‐distribution‐based limit error were ± 1.3 μm and ± 2 μm within 50 m, respectively, demonstrating that the proposed ranging system integrating pulse coarse with OFC achieved a μm‐level accuracy for 10‐m‐scale ranging tasks. More importantly, the proposed system can be further optimized in terms of active frequency stabilization, extended TOF range, and full‐wave sampling and processing. This study paves the way for effective long‐distance ranging with ultra‐high precision requirements.

## Author Contributions

**Cunliang Yang:** funding acquisition, writing – original draft, methodology. **Xiao Wang:** software, methodology. **Junhao Li:** data curation, validation. **Zihao Zhang:** data curation, investigation, validation. **Jingjing Wang:** validation, investigation. **Jinman Ge:** methodology, software. **Lizhong Xiao:** methodology, software, formal analysis. **Jijun Feng:** funding acquisition, writing – review and editing, methodology, conceptualization, supervision.

## Conflicts of Interest

The authors declare no conflicts of interest.

## Supporting information


Supporting Information S1

## Data Availability

The data that support the findings of this study are available from the corresponding author upon reasonable request.
