# Supplementary material for: A Ranging System Integrating Time‐of‐Flight Approach With Optical‐Frequency‐Comb Interferometry for Ultra‐Precise Long‐Distance Measurements
Source: Nanophotonics. 2026 Nov 11;15(22):e70343. doi: 10.1002/nap2.70343 (PMC13755749; doi:10.1002/nap2.70343)
Supplement: Supplementary file 1 [file NAP2-15-e70343-s001.docx]

Supporting information

**A ranging system integrating time-of-flight approach with pulse interference autocorrelation for ultra-precise long-distance measurements**

**Cunliang Yang ^a,^**^†^**, Xiao Wang ^b,^**^†^**, Zihao Zhang ^c^, Junhao Li ^a^, Jingjing Wang ^a^, Jinman Ge ^d^, Jijun Feng ^a,*^, Lizhong Xiao ^e,*^**

a Shanghai Key Laboratory of Modern Optical System, Engineering Research Center of Optical Instrument and System (Ministry of Education), School of Optical-Electrical and Computer Engineering, University of Shanghai for Science and Technology, Shanghai 200093, China
b Institute of Advanced Precision Spectroscopy of East China Normal University, Shanghai 200241, China
c National Key Laboratory of Electromagnetic Space Security, Tianjin 300308, China
d National Key Laboratory of Science and Technology on Space Microwave, China Academy of Space Technology, Xi’An 710199, China
e School of Computer Science and Information Engineering, Shanghai Institute of Technology, Shanghai 201418, China

† These authors contributed equally

*Corresponding author: fjijun@usst.edu.cn

Figure S1. Experimental setup for the hybrid TOF–OFC absolute ranging system.

Figure S2. Equipment of the hybrid TOF–OFC absolute distance measurement system.
